# Chronic Hypertonic Hyponatremia in Lung Cancer: The Utility of the Osmolar Gap

**DOI:** 10.7759/cureus.89100

**Published:** 2025-07-30

**Authors:** Yamin Thant, Oyinoluwapo Ogunbambi, Abigail E Hollingdale, Samson O Oyibo

**Affiliations:** 1 Internal Medicine, Peterborough City Hospital, Peterborough, GBR; 2 Oncology, Peterborough City Hospital, Peterborough, GBR; 3 Diabetes and Endocrinology, Peterborough City Hospital, Peterborough, GBR

**Keywords:** ethanol induced, hypertonic hyponatremia, lung cancer, osmolar gap, plasma osmolality

## Abstract

Hyponatremia is a frequent electrolyte disorder encountered in clinical practice. The differential diagnosis is vast and requires a methodical approach to determining the cause. Hypotonic hyponatremia is the commonest form and syndrome of inappropriate antidiuretic hormone secretion (SIADH) is a typical cause, especially in patients who have cancer. On the other hand, hyponatremia with elevated serum osmolality (hypertonic hyponatremia) is not common, and as such, clinicians are less familiar with this clinical scenario. Clinicians are even less familiar with the use of calculated osmolality and the osmolar gap in such scenarios. Therefore, it is easy to misdiagnose a patient with SIADH in the presence of cancer if a thorough biochemical analysis is not performed. We describe the case of a 56-year-old man who presented with episodes of dizziness and was demonstrated to have chronic hypertonic hyponatremia. Because of a previous diagnosis of mild SIADH several months prior, the presence of severe hyponatremia on a background of cancer, and relatively elevated urine osmolality, the elevated serum osmolality was not appreciated and the serum osmolar gap was not calculated. This resulted in a misdiagnosis of chronic SIADH. It was only after the osmolar gap was calculated and found to be significantly elevated that the markedly elevated serum ethanol level was discovered, and a diagnosis of alcohol-related hypertonic hyponatremia was made. On reassessment, the patient admitted to drinking large quantities of high alcohol-containing drinks for over the preceding three months. On gradual alcohol cessation, his blood results returned to normal. This case report highlights the importance of thorough assessment and the use of osmolar gap when navigating through the differential diagnosis of hyponatremia.

## Introduction

Hyponatremia is defined as a serum sodium level less than 135 mmol/L and is a frequent electrolyte disorder encountered in clinical practice. It is also associated with increased mortality, morbidity, and increased hospital length of stay. Hyponatremia can be classified as mild (130-134 mmol/L), moderate (125-129 mmol/L), and severe (<125 mmol/L) [1]. Common causes include medications, syndrome of inappropriate antidiuretic hormone secretion (SIADH), and underlying medical conditions such as heart failure, kidney disease, liver disease, hypothyroidism, Addison's disease, vomiting, and diarrhea. Most cases are asymptomatic, but symptoms can vary from mild, including nausea, vomiting, headache, and fatigue, to severe, including confusion, muscle weakness, seizures, and coma [1].

The differential diagnosis of hyponatremia is vast due to the multiple etiologies. The onset can be acute or chronic, the fluid status can be hypovolemic, euvolemic, or hypervolemic, the serum biochemistry can be hypo-osmolar (hypotonic), normo-osmolar (normotonic), or hyperosmolar (hypertonic), and the paired urine sodium concentration and osmolality can vary depending on the etiology. Most cases of hyponatremia are due to SIADH, where both the serum sodium and serum osmolality (measured and calculated) are less than normal, resulting in euvolemic hypotonic hyponatremia [2-4]. Hypertonic hyponatremia, on the other hand, is less common. These physiological and biochemical features form the basis of evidence-based guidelines for the management of hyponatremia. Thorough history taking, clinical examination and biochemical analysis is required to make a diagnosis and determine a treatment plan [2-4].

Hypertonic hyponatremia is caused by ingestion or administration of active osmolytes (e.g., alcohols, toxins, mannitol, glucose, etc). Despite the low serum sodium levels and low calculated osmolality, the laboratory-measured serum osmolality is elevated by the presence of these exogenous osmolytes. That is, the exogenous osmolytes are not included in the calculated osmolality but do increase the laboratory-measured osmolality. The resultant difference between the calculated and the laboratory-measured osmolality creates what is known as the osmolar gap [5-7].

The osmolar gap is a powerful diagnostic tool when trying to detect the presence of exogenous osmolytes in the serum during the investigation of hypertonic hyponatremia. An osmolar gap of more than 10 mOsm/kg should immediately prompt investigation for unmeasured osmolytes [5-7]. This simple calculation could prevent diagnostic delays and mismanagement in cases of hypertonic hyponatremia. Despite its importance, the osmolar gap is underutilized due to clinicians being unfamiliar with its use in clinical practice. Additionally, clinicians are more familiar with the clinical scenario of hypotonic hyponatremia rather than hypertonic hyponatremia. This dilemma results in diagnostic delays. We present a 56-year-old man with a three-month history of hypertonic hyponatremia. Because of previous biochemistry suggesting mild SIADH, the diagnosis of SIADH was still applied despite the presence of hyper-osmolality instead of the typical hypo-osmolality associated with SIADH. It was only after the hyper-osmolality and osmolar gap were appreciated that subsequent search discovered excess alcohol intake to be the cause of chronic hypertonic hyponatremia.

## Case presentation

Medical history and demographics

A 56-year-old man of Eastern European origin had three episodes of dizziness over a three-month period. Each episode was accompanied by a low serum sodium level (<120 mmol/L). He had no headache or seizure activity, no abdominal pain, vomiting or diarrhea, and no polydipsia or polyuria. His weight was stable. His medical history included stage-3B (non-squamous, non-small-cell) lung cancer diagnosed a year prior, dilated cardiomyopathy, coronary artery disease, type 2 diabetes, chronic kidney disease, chronic hepatitis B infection, and a previous partial gastrectomy. His medication list included combination chemoimmunotherapy (pemetrexed-pembrolizumab-carboplatin) every three weeks, metformin 1 gm twice a day, bisoprolol 5 mg daily, sacubitril/valsartan 24/26mg twice a day, isosorbide mononitrate 10 mg twice a day, glyceryl trinitrate spray as required, aspirin 75 mg daily, atorvastatin 80 mg daily, famotidine 20 mg daily, empagliflozin 10 mg daily, ivabradine 7.5 mg twice a day, spironolactone 25 mg daily, sertraline 50 mg daily, and modified release tamsulosin 400 mcg daily. He smoked 10-12 cigarettes daily and claimed that he did not drink much alcohol. His father died of lung cancer at a young age.

On examination, he was conscious and alert on each occasion. His blood pressure, heart rate, respiratory rate, and temperature were normal on all occasions. A thorough systems examination revealed no abnormal findings. His body mass index (BMI) was 19.1 kg/m^2^.

Investigations

Initial investigations demonstrated severe hyponatremia (123 mmol/L) with raised serum osmolality (333 mOsm/kg), indicating hypertonic hyponatremia. His thyroid hormones, stimulated cortisol, and liver enzymes were normal, respectively, ruling out hypothyroidism, hypoadrenalism, and liver impairment as causes of hyponatremia. He had mild macrocytic anemia with adequate serum vitamin B12 and folate levels (Table 1). His urine sodium concentration was low (<20 mmol/L) as opposed to high readings that are typical of SIADH. However, the urine osmolality was elevated for the degree of hyponatremia (Table 2). Historical results revealed a previous episode of hyponatremia several months prior with hypo-osmolality and raised urinary sodium and osmolality, which suggested mild SIADH.

**Table 1:** Initial blood test results. Demonstrating severe hypertonic hyponatremia with a significant osmolar gap.

Blood test	Result	Reference range
Sodium (mmol/L)	123	133-146
Potassium (mmol/L)	5.4	3.5-5.3
Chloride (mmol/L)	87	95-108
Creatinine (µmol/L)	60	59-104
Urea (mmol/L)	1.3	2.5-7.8
Glucose (mmol/L)	5.8	4-7
Thyroid-stimulating hormone (mU/L)	2.52	0.3-4.2
Calcium (mmol/L)	2.32	2.2-2.6
Measured serum osmolality (mOsm/kg)	333	275-295
Calculated serum osmolality (mOsm/kg)	253	275-295
Osmolar gap (mOsm/kg)	80	<10
Alanine transferase (IU/L)	15	<41
Aspartate transferase (IU/L)	41	10-50
Total bilirubin ((µmol/L)	5	0-21
Alkaline phosphatase (IU/L)	95	30-130
Albumin (g/L)	46	35-50
Globulin (g/L)	23	20-35
Total protein (g/L)	70	60-80
Pre-synacthen cortisol (nmol/L)	259	250-600
Post-synacthen cortisol (nmol/L)	769	>450
C-reactive protein (mg/L)	4	<10
Cholesterol (mmol/L)	5.0	<5.0
Triglycerides (mmol/L))	1.6	<1.7
Vitamin B12 (ng/L)	1397	200-771
Folate (µg/L)	>9.7	>3.0
Hemoglobin (g/L)	123	130-180
Mean corpuscular volume (fl)	100.8	80-100
White cell count (10^9^/L)	10.9	4.0-11.0
Platelet count (10^9^/L)	237	150-400

**Table 2:** Initial urine test results. Demonstrating low urine sodium as the body retains sodium in response to the low serum sodium levels.

Spot urine test	Result	Reference range
Osmolality (mOsm/kg)	226	300-1100
Sodium (mmol/L)	<20	40-220

Initially, the raised serum osmolality in the presence of hyponatremia was not appreciated. The severe hyponatremia with a urine osmolality above 100 mOsm/kg led the clinician towards SIADH. After consultation with the endocrinologist, the calculated serum osmolality value was obtained (253 mOsm/kg) and subtracted from the laboratory-measured serum osmolality to give the osmolar gap, which was markedly elevated (80 mOsm/kg; reference range: <10). The finding of hypertonic hyponatremia associated with a significant osmolar gap indicated the presence of unmeasured solutes or osmolytes in the serum. We then suspected excess alcohol intake, but the patient denied this. Therefore, ethanol concentration was assessed at his next appointment. The results demonstrated a high serum ethanol level of 36 mmol/L (reference range: <2.2 mmol/L) accompanied by a repeat measured osmolality of 315 mOsm/kg and a calculated osmolality of 265 mOsm/kg. On further questioning, the patient admitted that he drank excess alcohol daily in the form of spirits and high alcohol-containing beer, and that this was only in the preceding few months. However, he declined to elaborate on this further.

Treatment

The diagnosis of alcohol-related hypertonic hyponatremia was discussed with the patient during his regular oncology outpatient appointment. He was initially hospitalized so as to undergo an alcohol detox programme, which consisted of a reducing dose of chlordiazepoxide. He was also given vitamin B complex and discharged on oral thiamin 50 mg daily. He was also referred to the alcohol cessation team for advice regarding reducing alcohol consumption. He collected the alcohol cessation leaflet but did not attend for help. He also declined to have a session with the psychologist and the dietitian, as he wanted to self-manage.

Outcome and follow-up

**Figure 1:**
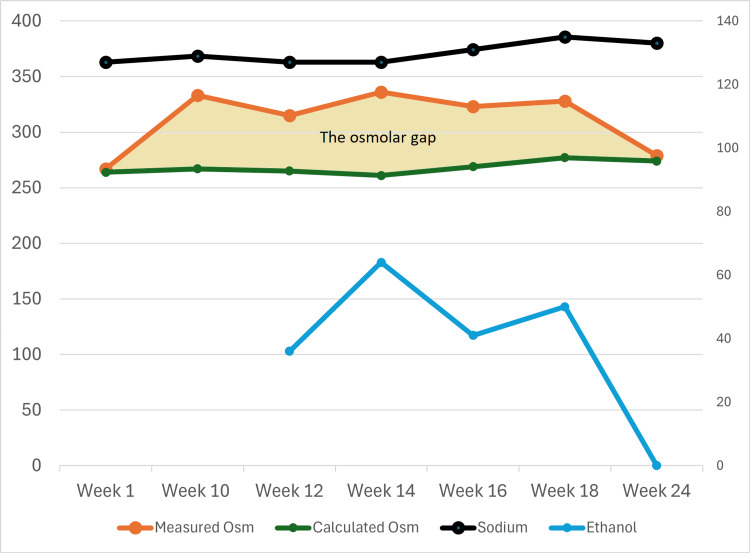
A series of the patient's serum ethanol, sodium, and osmolality values. The x-axis represents the weeks when blood tests were carried out; week 1: started drinking strong alcohol-containing drinks, week 12: with the aid of the osmolar gap, blood ethanol was detected, and week 24: stopped alcohol completely. The left y-axis represents the values for the measured and calculated serum osmolalities. The right y-axis represents the values for the serum ethanol and sodium concentrations. The blue line represents the serum ethanol values detected at week 12, gradually reducing and being undetectable at week 24. The black line represents the serum sodium values, which was low at week 1 and gradually climbed to normal by week 24, as the alcohol intake reduced. The green line represents the calculated serum osmolality, which mirrors the serum sodium values. The orange line represents the laboratory-measured serum osmolality, which was similar to the calculated osmolality at week 1, then elevated above the calculated osmolality during the ethanol intake, then went back down and became similar to the calculated osmolality on alcohol cessation at week 24. The light-yellow shaded area represents the osmolar gap.

The patient gradually reduced his alcohol intake until he stopped taking it altogether. Figure 1 demonstrates the progress of his serum ethanol, sodium, and osmolality levels and the osmolar gap during the six-month period, from when he started drinking strong alcohol-containing drinks to when he stopped. Blood results indicated the gradual reduction and final absence of ethanol in the bloodstream, corresponding with the absence of the osmolar gap and improvement of serum sodium levels. The patient remained under oncology follow-up.

## Discussion

We have reported the case of a man who had chronic severe hypertonic hyponatremia on a background of lung cancer. The background history of lung cancer, combined with a urine osmolality greater than 100 mOsm/kg in the presence of severe hyponatremia, led to a misdiagnosis of SIADH. The elevated laboratory-measured serum osmolality was not appreciated, and therefore, the calculated serum osmolality and the osmolar gap were not calculated. The observed urine osmolality would have been due to the presence of ethanol in the urine, producing a urinary osmolar gap akin to that occurring in the serum. However, we did not do a full urine profile. We believe the diagnostic delay was due to unfamiliarity with the clinical scenario of hypertonic hyponatremia and unfamiliarity with the calculation and use of the osmolar gap. Additionally, a previous history of mild SIADH co-contributed to a misdiagnosis of SIADH, despite the presence of a markedly elevated serum osmolality.

Hypertonic hyponatremia is a less common form of hyponatremia. There is a high concentration of exogenous osmotic substances (osmolytes) in the blood. These osmolytes draw water out of the cells, diluting the serum sodium (hyponatremia). However, the total body sodium is normal. Causes of excess osmolytes include intravenous glucose, intravenous immunoglobulin therapy, excessive salt and protein ingestion, intravenous administration of mannitol or glycine, and excess alcohol intake [2,3].

Serum osmolality is a function of the number of dissolved solutes per kilogram of serum. The main solutes (osmolytes) are sodium, chloride, bicarbonate, glucose, and urea [5]. Several formulae have been introduced for calculating the serum osmolality, but the Smithline-Gardner formula has been found to be the most fit for purpose [5-7]. This formula is designated as two multiplied by the concentration of sodium, then plus the concentration of glucose plus the concentration of urea, all in mmol/L ((2 x sodium) + glucose + urea), and the result is expressed as milliosmoles per kilogram of water (mOsm/kg H_2_O). The serum sodium multiplied by two takes account of the accompanying ions (chloride and bicarbonate). This formula does not account for any other exogenous osmolytes (e.g., ethanol, glycine, ethylene glycol, sorbitol, and methanol).

The measured serum osmolality, using a laboratory osmometer, gives us the true osmolality and takes into account the contribution from all solutes, including exogenous osmolytes. The calculated osmolality subtracted from the laboratory-measured osmolality gives us the osmolar gap. A normal osmolar gap is less than 10 mOsm/kg H_2_O, and an elevated or clinically significant osmolar gap is due to the contribution from the other osmolytes mentioned above. The actual contribution made by ethanol can be calculated as 1.25 multiplied by the serum ethanol concentration in mmol/L (1.25 x ethanol), and expressed as mOsm/kg H_2_O. This value should be equal to the osmolar gap if the osmolar gap is solely due to the presence of ethanol [6].

**Figure 2:**
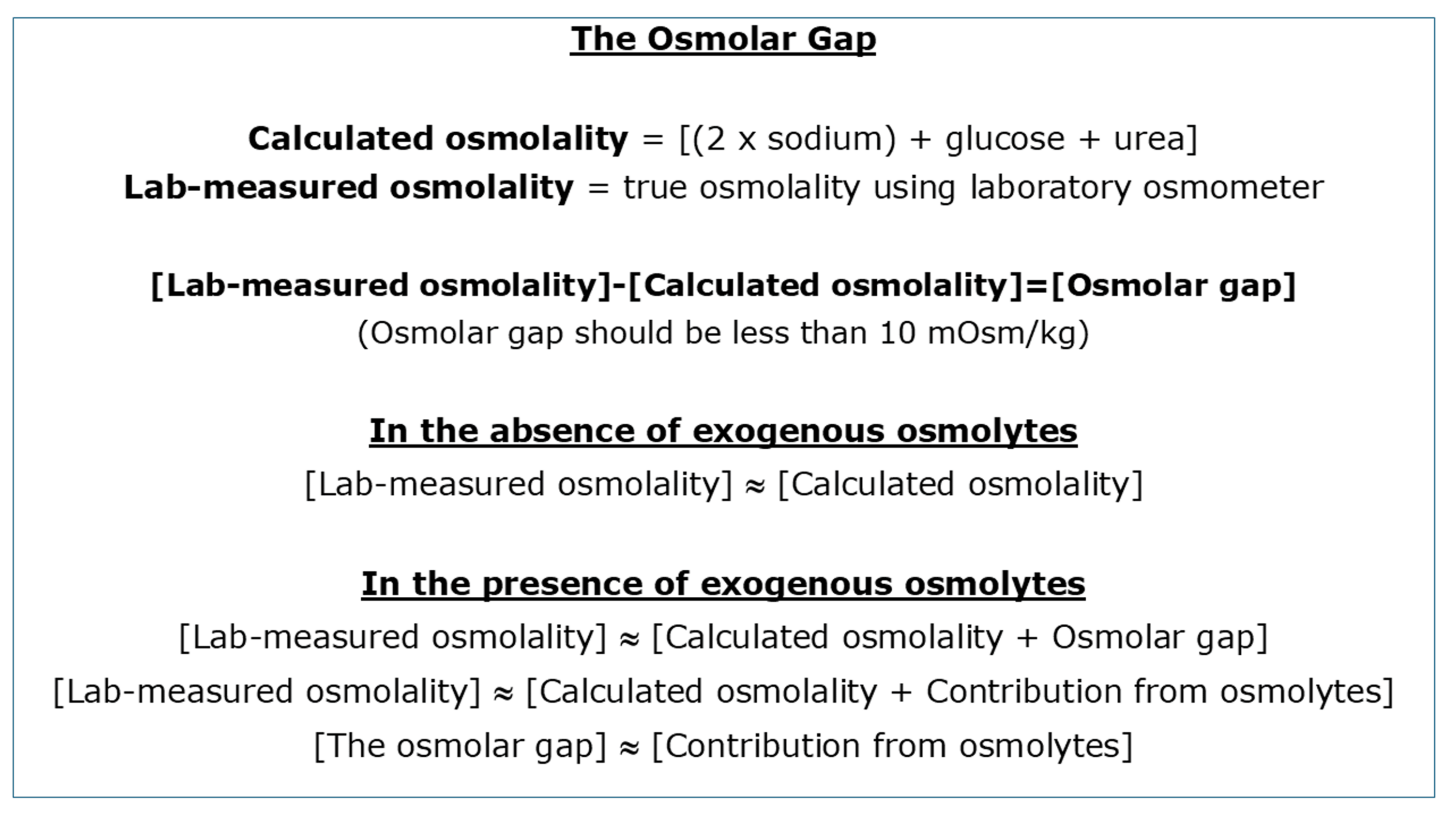
The osmolar gap. Lab: laboratory; the "approximately equal" sign is used where appropriate.

For example, on one occasion, this patient’s laboratory-measured serum osmolality was 315 mOsm/kg H_2_O, and the calculated osmolality was 265 mOsm/kg H_2_O, producing an osmolar gap of 50 mOsm/kg H_2_O. The serum ethanol concentration at the same time was 36 mmol/L. Therefore, the calculated contribution to the serum osmolality from ethanol was 45 mOsm/kg H_2_O (1.25 x 36). Therefore, ethanol was the osmolyte producing the observed osmolar gap. When the ethanol-related value is added to the calculated osmolality (45 + 265 = 310), the true calculated osmolality is similar to the laboratory-measured osmolality, with a difference of only 5 mOsm/kg (Figure 2).

Pseudohyponatremia is distinct from hypertonic hyponatremia. Pseudohyponatremia is due to a laboratory measurement artifact when the serum concentration is measured using the commonly used prior-dilution, indirect ion-specific electrode method (indirect ISE). The presence of certain endogenous substances (as opposed to exogenous substances) affects this analytical method through three main mechanisms: the electrolyte exclusion effect, the dilution effect, and the hyper-viscosity effect [8,9]. These endogenous substances include hyperproteinaemia, hypertriglyceridemia, hypercholesterolemia, and hyperglycemia due to their various medical conditions. Therefore, pseudohyponatremia is characterized by false hyponatremia and false low calculated osmolality but normal laboratory-measured osmolality, resulting in a false osmolar gap [8,9].

The main way to differentiate between true hyponatremia and pseudohyponatremia would be to obtain a sodium measurement using the point-of-care-testing (POCT) system, blood gas analyzers, or whole blood measurements, which all use the direct ISE method, as opposed to the indirect ISE method used in most laboratory systems [8,9]. Table 3 shows the causes of hyponatremia with a significant osmolar gap grouped under hypertonic hyponatremia and pseudohyponatremia.

**Table 3:** Causes of hyponatremia with a significant osmolar gap.

Hypertonic hyponatremia	Pseudohyponatremia
Ingestion of ethylene glycol, methanol, ethanol, isopropyl alcohol, formaldehyde, paraldehyde ingestion, and diethyl ether	Hyperproteinemia secondary to multiple myeloma, monoclonal gammopathies, Waldenström's macroglobulinemia, and HIV disease (hypergammaglobulinemia)
Intravenous infusion of non-conductive glycine, sorbitol, or mannitol solutions, and intravenous infusion of immunoglobulin	Hypertriglyceridemia secondary to pancreatitis, acute or chronic alcoholism, asparaginase treatment, diabetic ketoacidosis, poorly controlled type 2 diabetes, genetic defects (lipoprotein lipase), and lipoproteinemia (types I and V)
Hyperglycemia during hyperosmolar hyperglycemic state (HHS)	Hypercholesterolemia secondary to obstructive/cholestatic jaundice, pancreatic cancer with biliary obstruction, primary biliary cirrhosis, drug-induced cholestatic hepatitis, graft-versus-host liver disease, hepatitis, and genetic liver defect (e.g., Alagille syndrome)

Beer potomania is another differential diagnosis for hyponatremia in individuals who take excess alcohol. It is the occurrence of hypotonic hyponatraemia associated with excessive beer drinking [10]. Because beer is poor in salt and protein, excessive consumption and binge-drinking by alcoholics can result in dilutional hyponatraemia. Chronic alcoholics are generally malnourished with little salt or protein intake, such that an excessive water load results in low solute load to the kidneys, loss of the urea concentrating gradient in the kidneys, and inability of the kidneys to excrete free water. This results in severe dilutional (hypotonic) hyponatraemia followed by a hypotonic urine [11]. Our patient had hypertonic as opposed to hypotonic hyponatremia.

This patient had multiple comorbidities and polypharmacy. With a background history of lung cancer stable on chemotherapy and a previous finding of mild SAIDH, it is important that drug-induced hyponatremia is still ruled out, especially in a patient with renal impairment and diabetes mellitus. We believe this patient may have had underlying mild SIADH, which was replaced temporarily by alcohol-induced hypertonic hyponatremia.

This patient denied alcohol use at first, then admitted to drinking excessively when later questioned. His initial blood ethanol level was 36 mmol/L. Blood ethanol levels greater than 21.7 mmol/L can indicate intoxication or a potential for toxicity, and levels greater than 43.4 mmol/L can result in loss of consciousness. The true effects can vary depending on age, sex, body weight, and overall health. Additionally, habitual drinkers (chronic alcohol users) become tolerant and may not have any symptoms at high ethanol concentrations [12]. This case illustrates two important clinical points regarding excess alcohol usage: (1) patients may initially underreport or deny excess alcohol use, requiring tactful repeated questioning when clinical suspicion is high, and (2) chronic alcohol users may not exhibit obvious signs of intoxication despite dangerously high blood alcohol levels.

## Conclusions

Hyponatremia is a frequent electrolyte abnormality encountered during medical practice. The differential diagnosis is vast and requires a methodical approach. If the accompanying serum osmolality is normal or elevated, then the osmolar gap should be calculated. An osmolar gap greater than 10 mOsm/kg should immediately prompt investigation for unmeasured osmolytes like alcohols, toxins, or exogenous substances. This simple calculation could prevent diagnostic delays and mismanagement in similar cases.
